# COVID-19-Associated Coagulopathy: Ascending Aortic Thrombus

**DOI:** 10.7759/cureus.36607

**Published:** 2023-03-23

**Authors:** Maria E Mesalles, Shiavax J Rao, Usman Sagheer

**Affiliations:** 1 Internal Medicine, MedStar Union Memorial Hospital, Baltimore, USA

**Keywords:** arterial thrombus, ascending aortic thrombus, coagulopathy, aortic thrombus, coronavirus disease 2019, covid-19

## Abstract

The mechanism of arterial thrombosis in coronavirus disease 2019 (COVID-19) is not completely understood and is attributed to the complex interactions of endothelial injury, platelet hyperactivation, and activated pro-inflammatory cytokines. Management strategies may include a combination of surgery and anticoagulation, or anticoagulation alone. A 56-year-old woman with recent COVID-19 infection presented with chest pain and dyspnea. Chest CT angiography (CTA) and aortic magnetic resonance imaging revealed an intraluminal thrombus in the mid ascending aorta. A multidisciplinary team decided on heparin infusion. She was transitioned to apixaban and a three-month interval outpatient CTA revealed complete resolution of the aortic thrombus.

## Introduction

Coagulopathy is a well-established manifestation of severe coronavirus disease 2019 (COVID-19) infection. High incidence of thrombosis in hospitalized patients was identified early during the pandemic. The mechanisms of thrombosis in COVID-19 are not completely understood and are attributed to multiple complex interactions of endothelial injury, platelet hyperactivation, and activated pro-inflammatory cytokines [1].

The severity of COVID-19 is proportional to the risk of thrombotic complications, with rates of thrombosis being substantially high in hospitalized patients (~14%), and strikingly high in those that are critically ill (~40-57%) [2]. The prevalence of arterial thrombotic events is still not well understood, with retrospective data suggesting a prevalence of ~0.8-5.6% [2].

Most COVID-19 thrombotic complications are related to deep vein thrombosis and pulmonary embolism. Limited information is available regarding arterial thrombosis and only a few case reports describe intra-aortic thrombosis [3,4]. We present a unique and complex clinical case of a patient developing an ascending aortic thrombus (AAT) in the setting of COVID-19 infection.

This case was previously presented as a meeting abstract at the American College of Cardiology ACC22 National Meeting in April 2022.

## Case presentation

**Figure 1:**
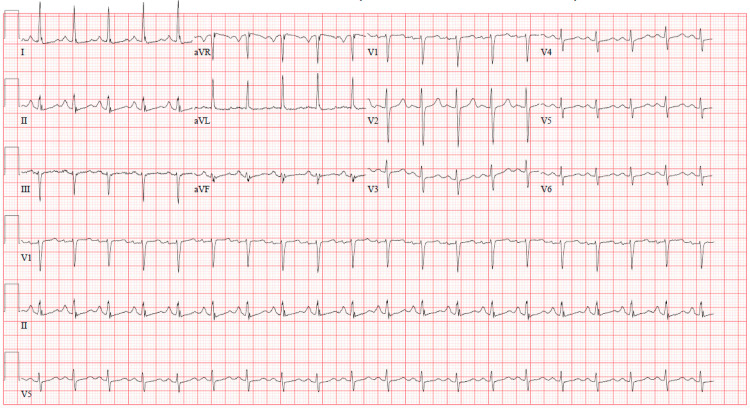
12-lead EKG revealing sinus tachycardia

A 56-year-old woman presented in the context of acute onset shortness of breath and chest pain. Of note, she had recently completed a two-week hospitalization for COVID-19 pneumonia, and was subsequently discharged to acute rehabilitation. Her medical history was significant for hypertension, type 2 diabetes mellitus, heart failure with improved ejection fraction (55 - 60%), hyperlipidemia, anxiety and gastroesophageal reflux disease. On this presentation (three weeks after her prior admission for COVID-19), she was tachycardic (119 beats/min), requiring 1 L supplemental oxygen via nasal cannula to maintain adequate oxygen saturations, with otherwise stable vital signs. Initial laboratory diagnostics including complete blood count and basic metabolic panel revealed mild hyponatremia (134 mmol/L; reference range 137-145 mmol/L) and anemia (hemoglobin 9.8 g/dL; reference range 11.0-14.15 g/dL). A 12-lead EKG confirmed sinus tachycardia (Figure 1).

**Figure 2:**
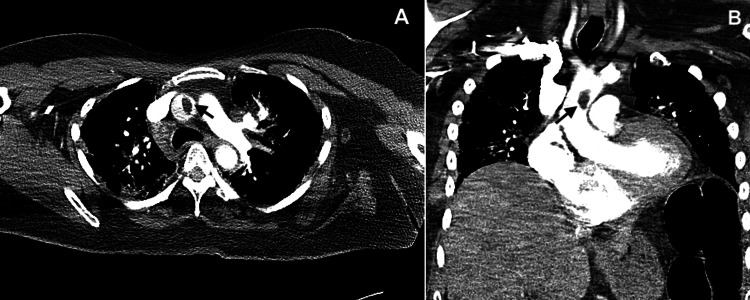
Computed tomography angiography of the chest, axial (A) and coronal (B) slices, showing an intraluminal filling defect (black arrows) in the mid ascending aorta.

Given concern for pulmonary embolism, stat CT angiography (CTA) of the chest was obtained. This did not show any evidence of pulmonary embolism, but did reveal a new 1.7 x 0.9 x 1.5 cm intraluminal defect within the ascending aorta, concerning for free-floating clot (Figure 2). 

**Figure 3:**
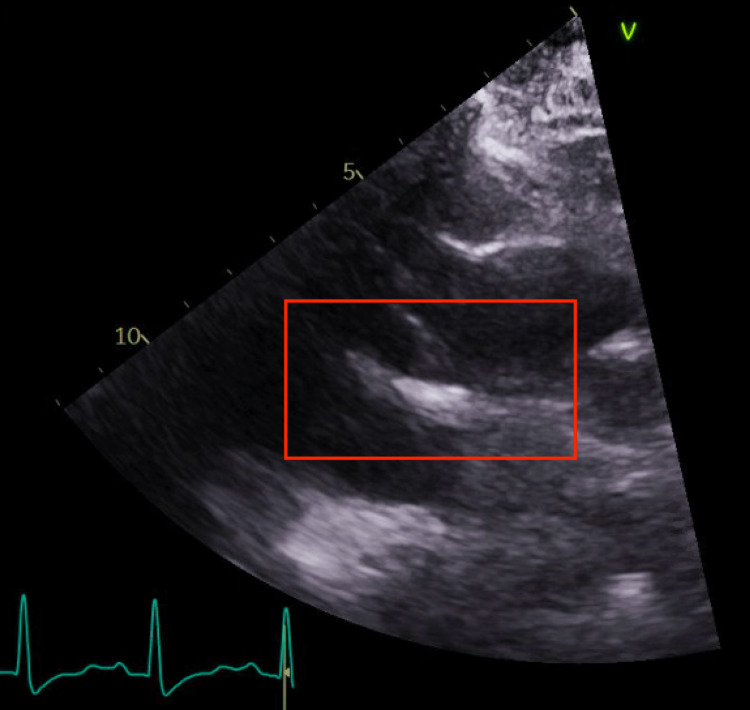
Transthoracic echocardiogram revealing a mobile linear echodensity in the aortic root

The vascular surgery and cardiology services were consulted for evaluation, and given concerns for embolization, the patient was initiated on a continuous infusion of heparin. A transthoracic echocardiogram was obtained, which revealed a mobile linear echodensity in the aortic root (Figure 3), without evidence of ventricular thrombus or endocarditis; however, the echodensity was ill-defined and the images were not definitive to corroborate or rule out an ascending aortic thrombus.

**Figure 4:**
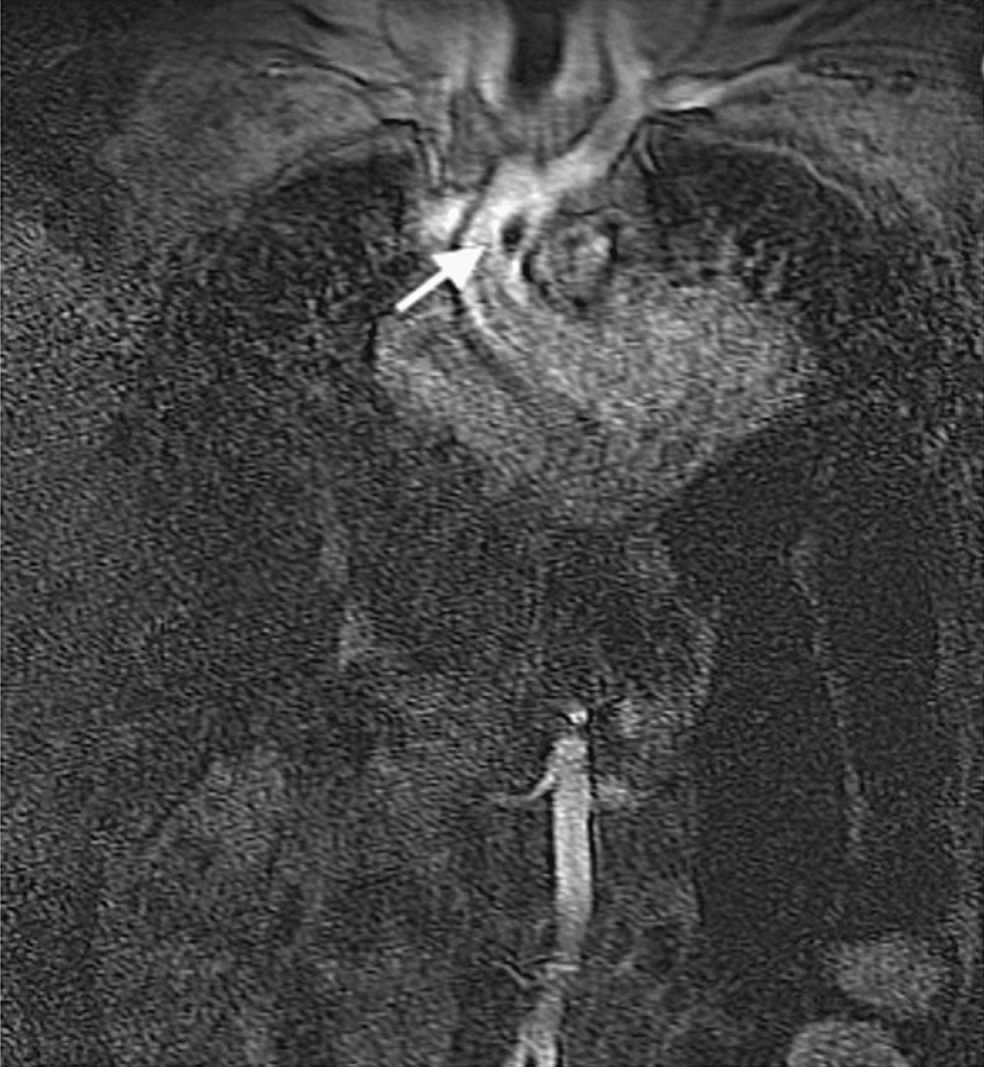
Contrast-enhanced magnetic resonance imaging showing an intraluminal filling defect (white arrow) in the mid ascending aorta with normal aortic size and wall thickness.

She subsequently underwent magnetic resonance angiography (MRA) of the chest which confirmed an intraluminal filling defect in the ascending thoracic aorta, most compatible with thrombus or an area of focal dissection (Figure 4). The remainder of the aorta as well as branch vessel origins were widely patent.

**Figure 5:**
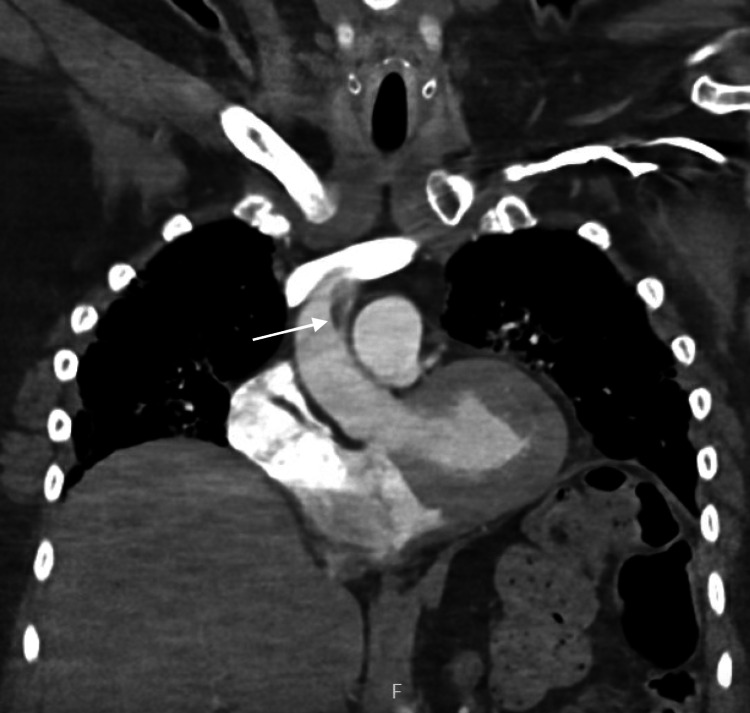
Ten-day interval chest CTA demonstrating a decrease in size of the aortic intraluminal filling defect

Given these findings, cardiothoracic surgery evaluated the patient for intervention for clot extraction. The recommendations from a multidisciplinary team approach included continuation of heparin infusion and repeating a 10-day interval CTA. This repeat study demonstrated a decrease in size of the aortic intraluminal filling defect (Figure 5).

The patient was transitioned from heparin infusion to apixaban, and discharged with planned outpatient follow-up. Complete resolution was noted on interval CTA of the chest after three months, which did not reveal any evidence of aortic dissection, aneurysm or thrombus.

## Discussion

Thromboembolic complications are commonly seen in critically ill patients with COVID-19, with a reported prevalence as high as 30% despite pharmacological prophylaxis [5]. The most frequently reported thromboembolic complications are deep vein thrombosis and pulmonary embolism arising from deep venous thrombosis and in situ immune-mediated pulmonary thrombosis. There is also an increased incidence of arterial thrombosis, especially cerebrovascular accidents and acute coronary syndromes. Coagulation activation is a specific feature distinguishing COVID-19 from other respiratory infections [5]. 

The coagulopathy seen in COVID-19 is characterized by high d-dimer and fibrinogen concentrations with minor changes in the platelet count, leading to a prothrombotic state [6]. It is a complex phenomenon and involves various dysregulated molecular pathways during the clinical progression of the disease. The respiratory tract invasion of SARS-CoV-2 results in a systemic inflammatory response with the consequent release of pro-inflammatory interleukins (IL) including IL-1, IL-6, IL-8, and tumor necrosis factor alpha. This activates coagulation leading to increased tissue factor expression, release of neutrophil extracellular traps, damage associated molecular patterns release, hyperfibrinogenemia, and increased thrombin generation. The resultant vascular inflammation leads to endothelial damage with subsequent deregulated coagulation activation [5,7,8]. Clinical studies have shown that patients with COVID-19 have higher levels of fibrinogen, fibrin degradation products, von Willebrand Factor and d-dimer levels, which appears to correlate with severity of infection and thrombotic risk [9]. Also, the renin-angiotensin-aldosterone system (RAAS) plays a role in COVID-19; SARS-CoV-2 uses angiotensin converting enzyme 2 (ACE2) to internalize within human cells, which may lead to a reduction in ACE2 activity, and a consequent an increase in angiotensin II, which has pro-inflammatory and prothrombotic effects [7,10].

Arterial thrombotic events, as well as microvascular thrombotic disorders, have been frequently documented in COVID-19 [6]. However, aortic thrombosis, specifically an ascending aortic thrombus in a non-aneurysmal aorta, is extremely rare due to the high blood flow and high pressures within the aorta [11]. Aortic thrombosis can have potentially catastrophic thromboembolic complications including renal infarction, stroke, and ischemic limbs [12-14]. In other circumstances, an aortic thrombus can be an incidental finding while looking for pulmonary embolism. In patients with COVID-19 with elevated d-dimer, the reported incidence of identifying an incidental aortic thrombus, while screening for pulmonary embolism with computed tomography angiography, is 0.75% [13].

The prevention and optimal management of thromboembolic events in COVID-19 remains a challenge. This is primarily due to an incomplete understanding of the underlying mechanisms of hypercoagulability, as well as the occurrence of thrombosis despite pharmacological thromboprophylaxis [8]. Management of ascending aortic thrombus with anticoagulation versus aortic surgery has been shown to result in similar outcomes [15]. Management strategies in patients with COVID-19 and ascending aortic thrombus can vary, including a combination of both surgical treatment and anticoagulation, or anticoagulation alone [12,14,16]. Our patient was started on therapeutic heparin, repeat imaging in 10 days showed decrease in size of thrombus, and therefore the patient was transitioned to apixaban with complete resolution of thrombus in three months.

## Conclusions

COVID-19 is a hypercoagulable and thrombogenic disease predisposing patients to both venous and arterial thrombosis and thromboembolic phenomena. Ascending aortic thrombosis is a rare phenomenon, the exact mechanism of which remains to be elucidated. Management strategies may include a combination of surgery and anticoagulation, or anticoagulation alone.
